# Unreliable association between self‐reported sense of direction and peripheral vestibular function

**DOI:** 10.1002/brb3.70000

**Published:** 2024-09-08

**Authors:** J. Gerb, T. Brandt, M. Dieterich

**Affiliations:** ^1^ Department of Neurology University Hospital, Ludwig‐Maximilians‐University Munich Germany; ^2^ Graduate School of Systemic Neuroscience Ludwig‐Maximilians‐University Munich Germany; ^3^ German Center for Vertigo and Balance Disorders University Hospital Ludwig‐Maximilians‐University Munich Germany; ^4^ Munich Cluster for Systems Neurology Munich Germany

**Keywords:** self‐report, self‐reported sense of direction, spatial memory, spatial orientation, vestibular system, vestibulopathy

## Abstract

**Background:**

Uni‐ or bilateral peripheralvestibular impairment causes objective spatial orientation deficits, which can be measured using pen‐and‐paper‐tests or sensorimotor tasks (navigation or pointing). For patients’ subjective orientation abilities, questionnaires are commonly used (e.g., Santa Barbara sense of direction scale [SBSODS]). However, the relationship between subjective assessment of spatial skills and objective vestibular function has only been scarcely investigated.

**Methods:**

A total of 177 patients (mean age 57.86 ± 17.53 years, 90 females) who presented in our tertiary Center for Vertigo and Balance Disorders underwent neuro‐otological examinations, including bithermal water calorics, video head impulse test (vHIT), and testing of the subjective visual vertical (SVV), and filled out the SBSODS (German version). Correlation analyses and linear multiple regression model analyses were performed between vestibular test results and self‐assessment scores. Additionally, groupwise vestibular function for patients with low, average, and high self‐report scores was analyzed.

**Results:**

Forty‐two patients fulfilled the diagnostic criteria for bilateral vestibulopathy, 93 for chronic unilateral vestibulopathy (68 unilateral caloric hypofunction and 25 isolated horizontal vestibulo‐ocular reflex deficits), and 42 patients had normal vestibular test results. SBSODS scores showed clear sex differences with higher subjective skill levels in males (mean score males: 4.94 ± 0.99, females 4.40 ± 0.94; Student's *t*‐test: t‐3.78, *p* < .001***). No stable correlation between objective vestibular function and subjective sense of spatial orientation was found. A multiple linear regression model could not reliably explain the self‐reported variance. The three patient groups with low, average, and high self‐assessment‐scores showed no significant differences of vestibular function.

**Conclusion:**

Self‐reported assessment of spatial orientation does not robustly correlate with objective peripheral vestibular function. Therefore, other methods of measuring spatial skills in real‐world and virtual environments are required to disclose orientation deficits due to vestibular hypofunction.

## Graphical abstract

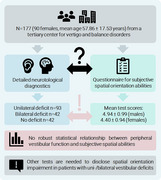

## 1 INTRODUCTION

Impairment of spatial orientation is a common symptom in a variety of disorders including peripheral vestibular hypofunction (Brandt et al., 2017; Burgess, 2008) and neurological diseases affecting cognition (Coughlan et al., 2018; Epstein et al., 2017; Segen et al., 2022). Since spatial orientation deficits can predate other cognitive symptoms in, for example, Alzheimer's disease (AD) by years (Coughlan et al., 2018), sensitive clinical tests of spatial skills are needed for early diagnosis. Recent research has therefore focused on the development of bedside tests as screening tests or potential progression markers (Allison et al., 2019, Levine et al., 2020). Different methods have been utilized to quantify spatial performance, for example, two‐dimensional (2D) tests (Kozhevnikov & Hegarty, 2001), virtual reality setups (Diersch & Wolbers, 2019; Kremmyda et al., 2016), real‐world navigation approaches (Schöberl et al., 2021; Schöberl et al., 2020), or three‐dimensional real‐world pointing tasks (3D‐RWPT, Gerb et al., 2022a; 2022b; Gerb et al., 2023, Gerb et al., 2024). All of these methods aim to quantify objective spatial orientation abilities.

Simple subjective assessments of spatial orientation are easy to obtain by self‐reports such as the Santa Barbara Sense of Direction Scale (SBSODS, Hegarty et al., 2002) or the Wayfinding Questionnaire (WQ, De Rooij et al., 2019). The SBSODS has been applied in multiple studies on spatial perception since 2002 (Hegarty et al., 2002; Hund & Padgitt, 2010; Meneghetti et al., 2014) (for an overview, see Cheng et al. [2022]). It includes 15 statements of subjective spatial abilities that participants answer on a 7‐point Likert scale. Age and sex effects on the overall score have been demonstrated repeatedly with male participants usually showing higher self‐report scores (Taillade et al., 2015). The questionnaire itself has a good test–retest reliability, but only moderate correlations with different behavioral navigation tasks. Similarly, age and sex differences have been described for the WQ (van der Ham et al., 2021) with older participants often overestimating their competence of spatial orientation.

Peripheral vestibular disorders are frequent (Brandt et al., 2022), often causing impairment of spatial orientation and navigation in the chronic stage (for a recent review, see Zwergal et al. [2023]). For example, bilateral peripheral vestibular hypofunction (bilateral vestibulopathy [BVP]) not only causes a dysfunction of spatial orientation in novel route combination (Schöberl et al., 2021). It has also been shown to induce a lack of self‐confidence (i.e., anxiety) to perform spatial navigation tasks, and can lead to hippocampal atrophy (Brandt et al., 2005; Kremmyda et al., 2016). Furthermore, vestibular hypofunction affects the selection of navigation strategies (Gammeri et al., 2022) and spatial performance in neuropsychological tests (Dordevic et al., 2021). Given the different therapeutical options for vestibular disorders on one hand and cognitive decline in dementia syndromes on the other hand, the correct differential diagnosis between the two has relevant clinical implications for management and rehabilitation of afflicted patients.

The meaningfulness of one's self‐assessment of spatial orientation competence is still under discussion. Nevertheless, the SBSODS is regularly applied in clinical routine. In a recent study on spatial orientation, we found no good correlation between the self‐reported sense of direction and objective performance in 2D and 3D spatial tests (Gerb et al., 2022b). Now, we analyzed the correlation between self‐reporting (SBSODS) and peripheral vestibular function measured by video head impulse testing (vHIT) and caloric irrigation. Recently, an investigation of the relationship between self‐reported sense of direction (using the SBSODS) and vestibular function assessed by vHIT and vestibular evoked myogenic potentials (VEMPs) in a cohort of 82 elderly healthy participants found a small number (*n* = 2) of patients with bilaterally impaired cervical VEMPs (cVEMPs) implying saccular hypofunction (Gandhi et al., 2021). These patients tended toward lower SBSODS scores in a multivariate linear regression model, the potential reason why a saccular dysfunction was postulated for navigational deficits in this elderly, otherwise healthy cohort. The authors concluded on the two exceptional cases that “self‐reported sense of direction appears to be associated with vestibular function” (Gandhi et al., 2021).

In order to test this hypothesis, in the current study on the investigation of potential correlations between objective tests of peripheral vestibular function and SBSODS scores, we included not only healthy elderly participants, but also younger patients and patients with chronic unilateral or bilateral vestibular hypofunction.

## 2 METHODS

A total of 177 patients (mean age 57.86 ± 17.53 years, 90 females) from our tertiary Center for Vertigo and Balance Disorders, Munich, Germany, with different neuro‐otological disorders underwent a detailed neurological and neuro‐otological workup including bithermal water calorics and vHIT, allowing for a state‐of‐the‐art assessment of peripheral vestibular function (Brandt et al., 2022). Furthermore, adjustments of the subjective visual vertical (SVV) were performed in order to assess vestibular graviceptive dysfunction and acute vestibular tone imbalances (Dieterich & Brandt, 1993). All patients were recruited for a clinical pointing task (3D‐RWPT); some had been included in previous studies on spatial perception (Gerb et al., 2022b; Gerb et al., 2023; Gerb et al., 2024). Most of the patients also underwent a cognitive screening test (Montreal Cognitive Assessment [MoCA]; Nasreddine et al., 2005).

The data protection clearance and Institutional Review Board of the Ludwig‐Maximilians‐University, Munich, Germany, approved the study (No. 094‐10). All patients gave informed consent. The study was performed in accordance with the ethical standards laid down in the 1964 Declaration of Helsinki and its later amendments.

### 2.1 Subjective self‐report of orientation abilities

All patients filled out a German version of the SBSODS (Hegarty et al., 2002), originally created for the validation study of the 3D‐RWPT (Gerb et al., 2022b) using the cross‐cultural adaptation process (Beaton et al., 2000). We calculated the final SBSODS score as instructed by the original test version (range 1–7, 1 = lowest score, 7 = highest score) and further defined three subsets: questions with an emotional component (e.g., “I don't enjoy giving directions.”), questions on subjective function (e.g., “I am very good at judging distances.”), and questions in which a higher score does not necessarily indicate better orientation abilities but possibly individual preferences (e.g., “I tend to think of my environment in terms of cardinal directions (N, S, E, W).”). Each item was classified as described in Gerb et al. (2024).

### 2.2 Neuro‐otological testing

Bithermal caloric testing was performed using warm (44°C) water irrigation in the right (WR) and left (WL) as well as cold (30°C) water irrigation in the right (CR) and left (CL) external ear canal to measure the function of the horizontal semicircular canals in the low‐frequency range of the vestibulo‐ocular reflex (VOR). Standardized vHIT measurements of the semicircular function were performed in the high‐frequency VOR range using the EyeSeeCamHIT system (EyeSeeTec, Munich, Germany). Caloric nystagmus was measured using video‐oculography infrared goggles and recorded according to convention, that is, positive values for left beating nystagmus and negative values for right‐beating nystagmus. Bilateral caloric deficits were defined as the total maximum slow phase velocity (SPV) of caloric nystagmus per side not exceeding 6°/s (according to the BáránySociety diagnostic criteria; Strupp et al., 2017) and caloric asymmetries were determined using Jongkees’ formula (cutoff value: 25%; Jongkees et al., 1962). For vHIT, a mean vHIT gain of <0.6 was considered pathological (Halmagyi et al., 2017)]. Furthermore, patients underwent a detailed neuroorthoptic assessment including measurement of the SVV to rule out acute vestibular tone imbalance using a spherical half dome. Only a small subcohort (*n* = 10) of patients underwent cervical and ocular VEMP testing, in order to diagnose or rule out superior canal dehiscence syndrome or to confirm complete vestibular loss in BVP.

Not all patients underwent all available vestibular tests, either due to contraindications (e.g., cervical injuries preventing vHIT testing or tympanic membrane perforation prohibiting water calorics). For example, some patients did not undergo all four paradigms of caloric testing when monothermal warm water calorics had already ensured intact bilateral peripheral vestibular function, resulting in a lower number of cold‐water recordings than warm water recordings. For these patients, Jongkees’ formula was not applicable and only their available SPV values were included in the correlation analyses. Furthermore, not all patients underwent MoCA testing. All patients with only partially available test results were excluded from the linear regression model.

### 2.3 Statistical analyses

We used JASP (JASP Team, 2023) to perform correlation analyses using Spearman's rho between the overall SBSODS test result, participant age, the three subsets (emotional, functional, and neutral), and the vestibular testing results (SPV of caloric nystagmus in °/s, VOR gain at 60 ms) as continuous variables, respectively. In a subsequent step, these correlation analyses were repeated for every single subitem of the SBSODS. Given the expected sex differences commonly shown in previous studies, statistical analysis was performed for the male and female patient cohort separate from one another. Additionally, a multiple linear regression model with vestibular testing results, patient age, and patient MoCA scores was created for the overall SBSODS score and the subsets. As a final analysis, sex‐specific SBSODS cutoff values were defined to divide each patient group into three evenly‐sized subgroups of low, average, and high self‐report scores, and age‐corrected analysis of covariance (ANCOVA) testing was performed to determine group differences in vestibular function.

## 3 RESULTS

**FIGURE 1:**
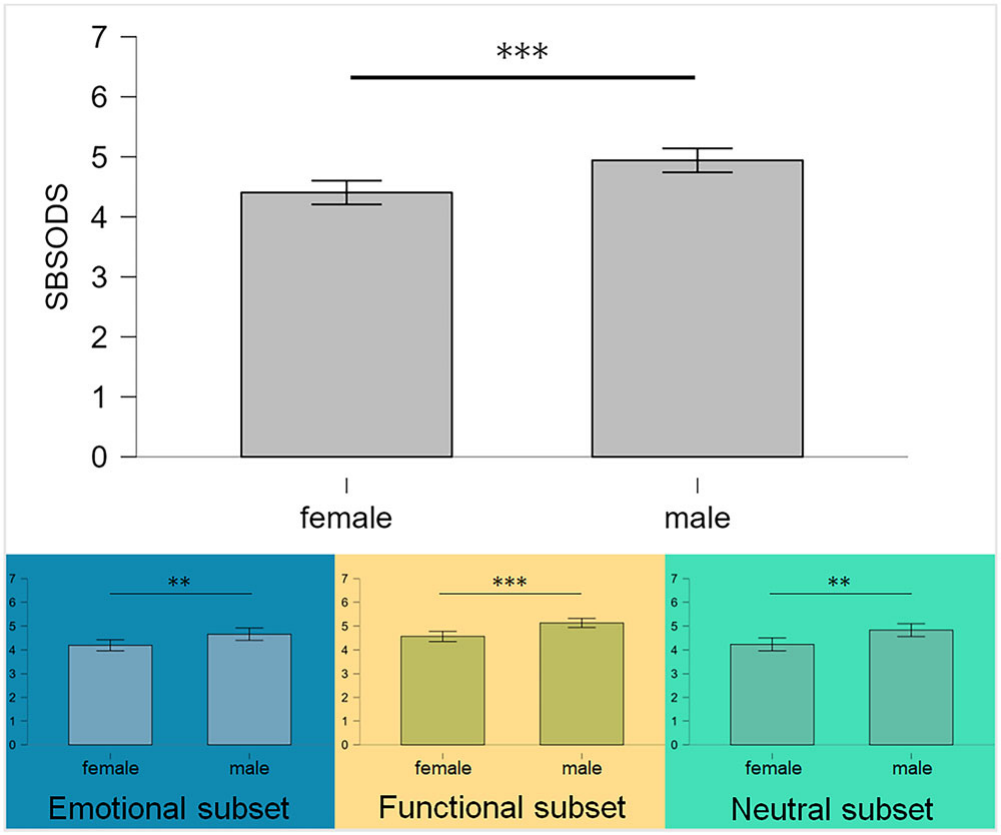
Bar plots of Santa Barbara sense of direction scale (SBSODS) scores divided by participant sex, showing a clear gender difference with males typically reporting higher scores (= better self‐reported sense of direction). This pattern is also visible in the three subsets defined by questions with an emotional component, questions on self‐assessed function, and questions that do not necessarily imply better or worse performance but rather individual preferences (smaller plots, blue: emotional subset; yellow: functional subset; green: neutral subset).

An independent samples Student's *t*‐test confirmed the expected sex differences with male participants typically reporting higher subjective assessments of direction in the SBSODS (mean score males: 4.94 ± 0.99, females 4.40 ± 0.94; emotional subset: males 4.66 ± 1.28, females 4.20 ± 1.11, functional subset males 5.14 ± 0.96, females 4.57 ± 1.02, neutral: males 4.83 ± 1.35, females 4.23 ± 1.30; Student's *t*‐test: overall SBSODS score t‐3.78, *p* < .001***, emotional subset: t‐2.62, *p* = 9.59 × 10^−3^**, functional subset t‐3.91, *p* < .001***, neutral subset t‐3.07, *p* = 2.43 × 10^−3^**; Figure 1).

In male patients, the overall score correlated positively with age (Spearman's rho 0.23, *p* = .02*); in female patients, no correlation was found (Spearman's rho −0.12, *p* = .25). A moderate positive age effect was found in male participants for the neutral subset (Spearman's rho 0.33, *p* < .001***) but not in the other subsets (Spearman's rho age/emotional subset 0.13, *p* = .21, age/functional subset 0.13, *p* = .19). For female participants, a weak negative age effect on the emotional subset was observable (Spearman's rho −0.22, *p* = .04*) but no effect in the other subsets (Spearman's rho age/functional subset −0.14, *p* = .20, age/neutral subset 0.11, *p* = .30).

**FIGURE 2:**
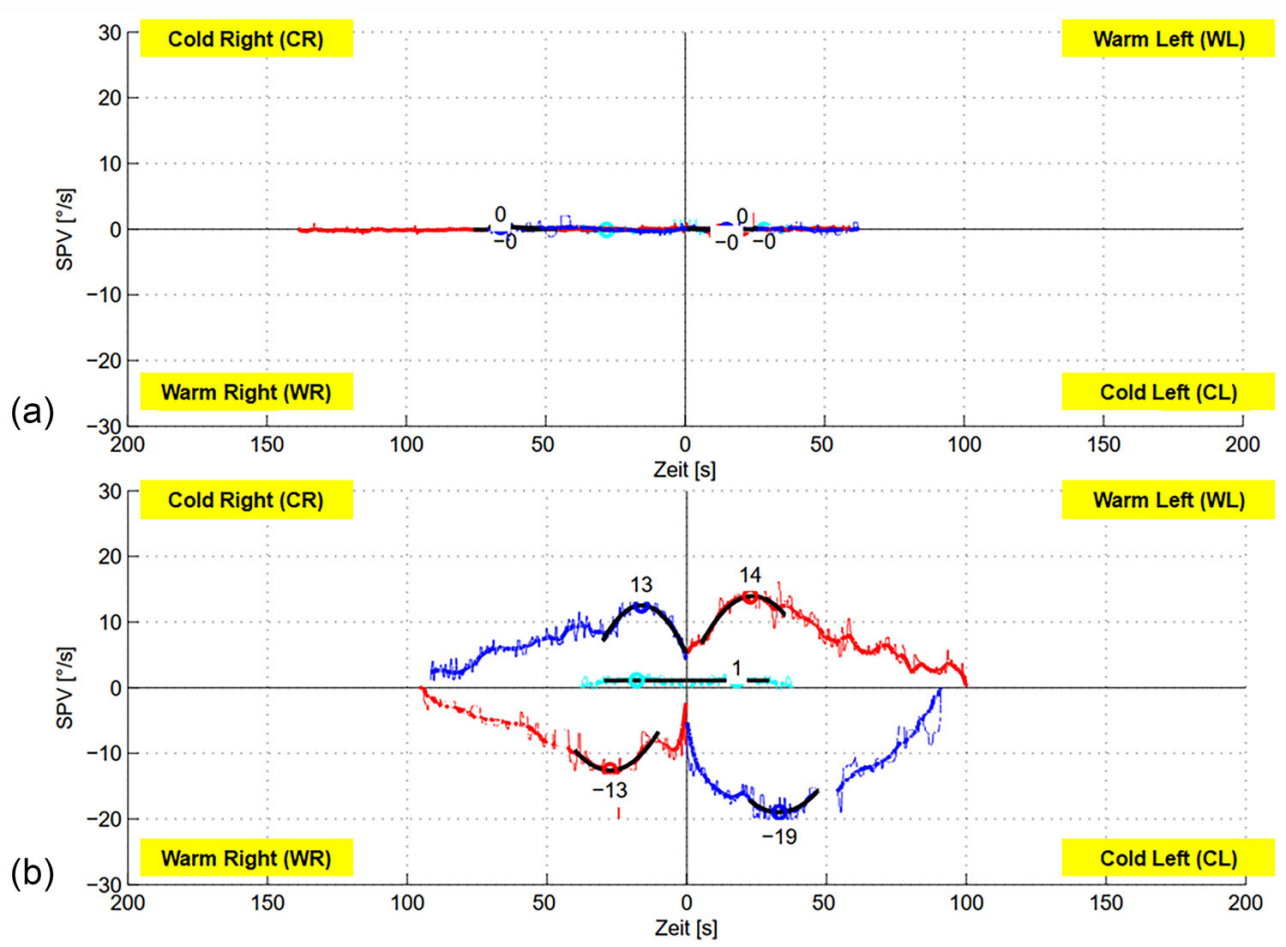
Examples of caloric test results from two patients (a and b) with an identical Santa Barbara Sense of Direction Scale (SBSODS) score of 5.0 pts (emotional subset: 4.2 pts, 4.4 pts; functional subset: 4.9 pts, 4.6 pts; neutral subset: 5.3 pts, 6.3 pts). Vestibular testing revealed a complete bilateral vestibulopathy in patient (a), whereas patient (b) showed normal peripheral vestibular function in bithermal calorics. a: male patient, 62.3 years; b: female patient, 82.3 years; blue lines: caloric nystagmus slow phase velocity (SPV) in °/s during cold water (30°C) irrigation; red lines: caloric nystagmus SPV in °/s during warm water (44°C) irrigation. The left patient side is plotted on the right diagram side.

*Vestibular testing* revealed complete BVP according to diagnostic criteria by the Bárány Society (Strupp et al., 2017) in 42 patients (14 females; Figure 2). A unilateral caloric hyporesponsiveness (caloric asymmetry index > 25%) was found in 68 patients (37 females). VHIT testing showed isolated unilateral high‐frequency deficits (gain < 0.6 on either left or right side) in 25 patients (12 females). In 31 patients, an SVV tilt (17 to the left, 14 to the right; mean absolute deviation 3.57° ± 1.23°, maximum deviation: 7° in a patient with incomplete central compensation of an acute unilateral vestibulopathy 9 months prior to presentation) was found, while 133 patients had no pathological SVV deviation. oVEMP recordings from a small subcohort (*n* = 10) revealed bilateral utricular hypofunction in three patients. cVEMPs (*n* = 10) showed bilateral dysfunction in two patients, unilateral saccular dysfunction in two patients, and normal findings in six patients.

Vestibular function was not normally distributed, as was expected due to our study design including patients from the outpatient dizziness clinic with manifest deficits (Shapiro–Wilk test: WR 0.85, *p* < .001***; WL 0.89, *p* < .001***; CR 0.90, *p* < .001***; CL 0.92, *p* < .001***; vHIT R 0.98, *p* = 5.86 × 10^−3^**; vHIT L 0.96, *p* < .001***).

**FIGURE 3:**
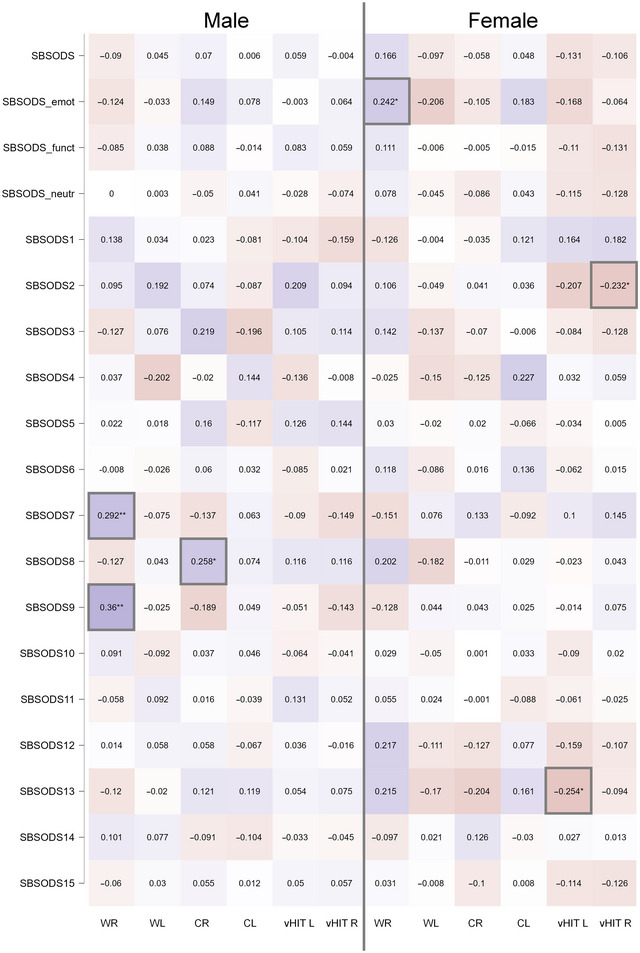
Heatmap of correlations (Spearman's rho) between Santa Barbara Sense of Direction Scale (SBSODS) overall scores (SBSODS, first row), subset scores (second row: emotional subset, third row: functional subset, fourth row: neutral subset) and individual items (following rows) and vestibular function diagnostics (bithermal calorics with warm water (44°C) caloric irrigation on the right (WR) and the left side (WL), cold water (30°C) caloric irrigation on the right (CR) and left side (CL), video head impulse test gain at 60 ms for the right [vHIT R] and the left side [vHIT L]), divided by patient sex. Significant correlations are marked with gray boxes.

***Statistical analysis*** of objective peripheral vestibular function and subjective self‐reported assessment of orientation was performed for the available vestibular test results (calorics: WR 160 patients, 81 females; WL 159 patients, 80 females; CR 152 patients, 75 females; CL 151 patients, 74 females; vHIT [L + R] 162 patients, 81 females). Correlation analysis using Spearman's rho found no stable correlation between overall SBSODS score and vestibular function. In the subset analysis, a weak correlation between WR (warm water irrigation of the right auditory canal) and the emotional subset was observable in female participants (Spearman's rho 0.24, *p* = .03*). None of the other subsets or other vestibular testing results reached statistical significance. In the subsequent single‐item analysis, no clear link between vestibular function and the respective item was seen, while some subitems showed weak partial correlations (male participants: WR and question 7: “I enjoy reading maps,” Spearman's rho 0.29, *p* = 9.14 × 10^−3^**; WR and question 9: “I am very good at reading maps,” Spearman's rho 0.36, *p* = 1.13 × 10^−3^**, CR and question 8: “I have trouble understanding directions,” Spearman's rho 0.26, *p* = .02*; female participants: vHIT L and question 13: “I usually let someone else do the navigational planning for long trips,” Spearman's rho −0.25, *p* = .02*, vHIT R and question 2: “I have a poor memory for where I left things,” Spearman's rho −0.23, *p* = .04*). A heatmap of all correlations can be found in Figure 3 and in Table S1.

For the multiple linear regression model, a total of 109 patients were included who had undergone all sets of measurements (vHIT, bithermal water calorics, MoCA; mean age 59.41 ± 15.94, 52 females). With the SBSODS as the dependent variable, using patient sex as a factor and including WR, CR, WL, CL, vHIT R, vHIT L, patient age, and MoCA‐score as covariates, no significant model was found (*F*(9,99) = 1.45, *p* = .18, *R* ^2^ = 0.12). Only for the emotional subset, statistical significance was reached (*F*(9,99) = 2.09, *p* = .04*, *R* ^2^ = 0.16) while the resulting model had a very weak prognostic value, only explaining 16% of the variance. Neither for the functional subset (*F*(9,99) = 1.73, *p* = .09, *R* ^2^ = 0.14) nor for the neutral subset (*F*(9,99) = 1.22, *p* = .29, *R* ^2^ = 0.10) significant models were found. For all models, case wise diagnostics showed no cases where the absolute standard residual value exceeded 3 (i.e., no relevant outliers), and the Durbin–Watson test always gave values between 1 and 3, therefore ruling out autocorrelation (overall SBSODS: 2.16, emotional subset: 2.14, functional subset: 2.10, neutral subset: 2.17).

**FIGURE 4:**
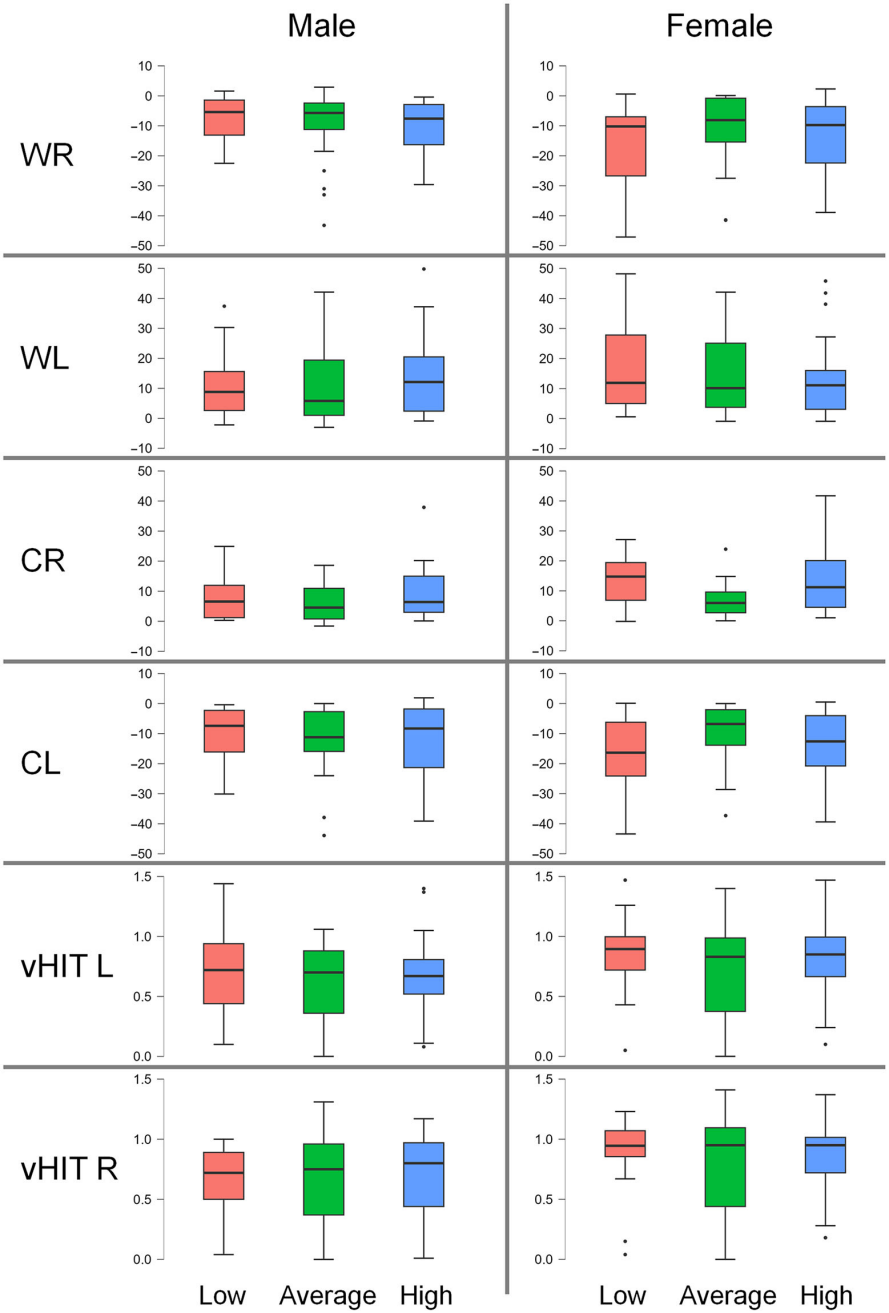
Mean vestibular testing results for evenly sized groups with low (red boxplots), average (green boxplots), and high (blue boxplots) Santa Barbara Sense of Direction Scale (SBSODS) scores, divided by patient sex (cutoff values for male participants: low self‐estimated sense of direction group: SBSODS < 4.4, average: SBSODS between 4.4 and 5.5, high: SBSODS > 5.5; females: low self‐estimated sense of direction group: SBSODS < 4.0, average: SBSODS between 4.0 and 4.8, high: SBSODS > 4.8). For WR (warm caloric irrigation of the right side) and CL (cold caloric irrigation of the left side), negative values for the nystagmus slow phase velocity (in °/s) were to be expected. In all diagnostic tests, values close to 0 imply severe hypofunction. All groups, regardless of low, average, or high self‐reported sense of orientation, showed similar vestibular testing results, and analysis of covariance (ANCOVA) testing (corrected for patient age) of group differences only reached statistical significance once (cold caloric irrigation of the right side [CR] in female patients, *F*(2,71) = 4.36, *p* = .02*), but in no other constellation. vHIT L, video head impulse test for the left side; vHIT R, video head impulse test for the right side; WL, warm caloric irrigation of the left side; black dots: outliers.

Additionally, the patients were divided into three equally large subgroups of low, average, and high self‐estimated spatial competence. The cutoff values were calculated individually for male and female participants (male participants: low self‐estimated sense of direction group: SBSODS < 4.4, average: SBSODS between 4.4 and 5.5, high: SBSODS > 5.5; females: low self‐estimated sense of direction group: SBSODS < 4.0, average: SBSODS between 4.0 and 4.8, high: SBSODS > 4.8), and age‐corrected ANCOVA testing was performed to detect group differences in the respective vestibular testing results. Again, no robust effect of the grouping variable was observable (male patients: WR: *F*(2,77) = 0.55, *p* = .58; WL: *F*(2,75) = 1.26, *p* = .29; CR: *F*(2,73) = 2.73, *p* = .07; CL: *F*(2,73) = 0.34, *p* = .71; vHIT R: *F*(2,77) = 0.84, *p* = .44; vHIT L: *F*(2,77) = 0.61, *p* = .55; female patients: WR: *F*(2,77) = 2.85, *p* = .06; WL: *F*(2,76) = 1.14, *p* = .33; CR: *F*(2,71) = 4.36, *p* = .02*; CL: *F*(2,70) = 2.35, *p* = .10; vHIT R: *F*(2,77) = 1.25, *p* = .29; vHIT L: *F*(2,77) = 0.85, *p* = .43; Figure 4).

An independent sample Student's *t*‐test showed no differences of the self‐report scores between patients with normal SVV and pathological SVV deviations (mean SBSODS score in patients with normal SVV: 4.66 ± 0.96, mean SBSODS score in patients with pathological SVV‐deviation 4.68 ± 0.98; emotional subset: patients with normal SVV: 4.39 ± 1.15, patients with SVV‐deviation 4.41 ± 1.33; functional subset: patients with normal SVV: 4.81 ± 1.02, patients with SVV‐deviation 4.90 ± 0.89; neutral: patients with normal SVV: 4.59 ± 1.34, patients with SVV‐deviation 4.58 ± 1.32; Student's *t*‐test: overall SBSODS score t‐0.11, *p* = .91, emotional subset: t‐0.09, *p* = .93, functional subset: t‐0.42, *p* = .67, neutral subset: t‐0.11, *p* = .91).

With only a small number of c/oVEMP recordings available (*n* = 10), no meaningful statistical analysis was possible. For the oVEMP recordings, patients with bilateral hypofunction (*n* = 3) had slightly lower SBSODS scores (mean score normal oVEMPs: 4.98 ± 0.77, mean score pathological oVEMPs 3.84 ± 0.91, emotional subset: normal oVEMPs: 4.23 ± 0.80, pathological oVEMPs 4.00 ± 0.72, functional subset normal oVEMPs: 5.00 ± 0.91, pathological oVEMPs 3.48 ± 1.19, neutral: normal oVEMPs: 5.29 ± 0.97, pathological oVEMPs 4.11 ± 0.96). Note that the patients with bilateral oVEMP pathologies included more females (*n* = 3, two females), whereas the patients with normal oVEMP recordings were predominantly male (*n* = 7, one female); given the significant sex differences in SBSODS scores, this might constitute a relevant confounder.

For the cVEMPs, patients with bilateral hypofunction (*n* = 2) or unilateral hypofunction (*n* = 2) again showed slightly lower SBSODS scores (mean score normal cVEMPs: 4.86 ± 0.77, mean score unilaterally pathological cVEMPs 4.27 ± 2.07, mean score bilaterally pathological cVEMPs 4.37 ± 0.05, emotional subset: normal cVEMPs: 4.13 ± 0.83, unilaterally pathological cVEMPs 4.00 ± 1.13, bilaterally pathological cVEMPs 4.40 ± 0.28, functional subset normal cVEMPs: 4.95 ± 0.99, unilaterally pathological cVEMPs 3.71 ± 2.22, bilaterally pathological cVEMPs 4.14 ± 0.40, neutral: normal cVEMPs: 5.06 ± 0.83, unilaterally pathological cVEMPs 4.83 ± 2.59, bilaterally pathological cVEMPs 4.67 ± 0.00). Again, the patients with normal oVEMP recordings (*n* = 6, one female) were predominantly male compared to the evenly distributed subgroups (unilateral: *n* = 2, one female; bilateral: *n* = 2, one female). All patients with unilateral or bilateral deficits in the o/cVEMP tests showed further deficits in the other diagnostic tests and fulfilled the diagnostic criteria for BVP.

## 4 DISCUSSION

In the current study, no robust association was observable between subjective sense of orientation and objective vestibular test results in a diverse cohort of patients with unilateral or bilateral vestibular hypofunction as well as patients without a vestibular deficit. In our patient cohort, a clear sex difference (with male patients exhibiting higher self‐report scores) was observable, similar to previous findings from studies on self‐reported orientation abilities in participants (Meneghetti et al., 2014; Schinazi et al., 2023; van der Ham et al., 2021) in whom a vestibular impairment was not searched for. Additionally, we found moderate positive age effects in male patients (higher scores in older patients) and moderate negative age effects on the emotional subset in female patients (lower scores in older patients).

Overall, the self‐reported SBSODS scores in the German version from patients with various neurotological disorders were comparable to other studies, for example, the validation work by Hegarty et al. (2002), who reported a mean score of 4.7 ± 1.1 in 82 participants, or by Schinazi et al. (2023) (*N* = 60, median score 4.93 for males, 4.07 for females), underlining the general validity of the German version and its applicability in disorders of vestibular function.

However, the SBSODS scores could not predict normal unilateral or bilateral peripheral vestibular function or vestibular deficits. This is only partially in line with Gandhi et al. (2021), who reported similar negative results for vHIT gain and SBSODS scores in their participant cohort but described a significant influence of suspected bilateral utricular dysfunction (determined by “oVEMP irreproducibility”) on the SBSODS scores. Given that our cohort only included a small subcohort with cVEMP and oVEMP recordings, a conclusive evaluation of VEMPs in this context is inappropriate. However, the clinical significance of isolated utricular or saccular dysfunction or its impact on everyday clinical diagnostics is questionable, especially when considering the known methodological pitfalls in VEMP diagnostics (Dlugaiczyk, 2020; Ertl et al., 2016). In our patient cohort, all patients with oVEMP or cVEMP pathologies fulfilled the diagnostic criteria of BVP, according to the bilateral pathological results in caloric and HIT testing of both ears. Given our study setting in an outpatient clinic for patients with vertigo and balance disorders, a higher rate of vestibular dysfunction compared to the normal population was expected, which was confirmed by the vestibular functional diagnostics. The simple use of SBSODS was unreliable in predicting the peripheral vestibular deficits found in detailed neuro‐otological investigations in our study on 177 dizzy patients.

It is unclear whether the discrepancy between the subjective sense of spatial direction and objective vestibular test results stems from patients being unaware of their spatial impairment due to vestibular hypofunction or whether vestibular spatial impairment affects other domains than the ones assessed in the SBSODS. The first assumption is unlikely since the missing correspondence was also evident in patients without vestibular deficits. Interconnections between the vestibular system and emotional processing might constitute a relevant confounder (Lopez, 2016). Intact vestibular function is required to perceive vertigo‐related anxiety in dizzy patients (Decker et al., 2019), and it is possible that patients with unilateral or bilateral vestibular hypofunction exhibit an altered emotional response to the imaginary situations assessed in the SBSODS, for example, getting lost in a new environment or having to remember a novel route. This could result in a skewed self‐report of these situations.

## 5 CONCLUSION

In our patient cohort with peripheral vestibular hypofunction of varying degree and normal peripheral vestibular function, no stable relationship between objective vestibular function and subjective sense of direction was found. Using the SBSODS as a screening tool for navigational deficits in (suspected or factual) vestibular dysfunction is therefore not recommended. The self‐assessment of spatial orientation and navigation is dependent on a subjective comparison with the general population and the life‐long experience of these sensorimotor tasks. This means that the questionnaire interrogates these abilities retrospectively, while the clinical tests reflect the actual performance. The current version of SBSODS does not include questions on age‐dependent changes of spatial orientation competence. Other methods for measuring spatial abilities in vestibular hypofunction should be chosen, for example, behavioral real‐world tests, which ideally include physiological vestibular stimuli.

## AUTHOR CONTRIBUTIONS

**J. Gerb**: Conceptualization; investigation; writing—original draft; methodology; validation; writing—review and editing; data curation; visualization; software. **T. Brandt**: Conceptualization; supervision; writing—review and editing; resources; validation. **M. Dieterich**: Conceptualization; funding acquisition; supervision; project administration; writing—review and editing; resources; validation.

## CONFLICT OF INTEREST STATEMENT

The authors declare no conflicts of interest.

### PEER REVIEW

The peer review history for this article is available at https://publons.com/publon/10.1002/brb3.70000.

## Supporting information

Supporting Information

## Data Availability

The data that support the findings of this study are available on request from the corresponding author. The data are not publicly available due to privacy or ethical restrictions.
