# Supplementary material for: Unreliable association between self‐reported sense of direction and peripheral vestibular function
Source: Brain Behav. 2024 Sep 8;14(9):e70000. doi: 10.1002/brb3.70000 (PMC11381552; doi:10.1002/brb3.70000)
Supplement: Supplementary file 1 — Supporting Information [file BRB3-14-e70000-s001.docx]

# Suppl. Table 1

Spearman’s correlation (rho) between vestibular function diagnostics (bithermal calorics with warm water (44°C) caloric irrigation on the right (WR) and the left side (WL), cold water (30°C) caloric irrigation on the right (CR) and left side (CL), video head impulse test gain at 60ms for the right (vHIT R) and the left side (vHIT L)) and SBSODS overall scores, subset scores, and individual questions, all divided by patient sex. Significant correlations are marked (* p<0.05, ** p<0.01).

| Spearman's correlations | |  | Male patients | |  |  | Female patients | |  |  |
| --- | --- | --- | --- | --- | --- | --- | --- | --- | --- | --- |
|  |  |  | **n** | **Spearman's rho** | | **p** | **n** | **Spearman's rho** | | **p** |
| WR | - | SBSODS | 79 | -0.09 |  | 0.43 | 81 | 0.17 |  | 0.14 |
| WR | - | SBSODS_emot | 79 | -0.12 |  | 0.28 | 81 | 0.24 | * | 0.03 |
| WR | - | SBSODS_funct | 79 | -0.09 |  | 0.45 | 81 | 0.11 |  | 0.32 |
| WR | - | SBSODS_neutr | 79 | -46.8 ×10^-3^ |  | 1 | 81 | 0.08 |  | 0.49 |
| WR | - | SBSODS1 | 79 | 0.14 |  | 0.22 | 81 | -0.13 |  | 0.26 |
| WR | - | SBSODS2 | 79 | 0.09 |  | 0.4 | 81 | 0.11 |  | 0.35 |
| WR | - | SBSODS3 | 79 | -0.13 |  | 0.26 | 81 | 0.14 |  | 0.21 |
| WR | - | SBSODS4 | 79 | 0.04 |  | 0.75 | 81 | -0.02 |  | 0.83 |
| WR | - | SBSODS5 | 79 | 0.02 |  | 0.85 | 81 | 0.03 |  | 0.79 |
| WR | - | SBSODS6 | 79 | -80.4  ×10^-3^ |  | 0.95 | 81 | 0.12 |  | 0.29 |
| WR | - | SBSODS7 | 79 | 0.29 | ** | 9.14×10^-3^ | 81 | -0.15 |  | 0.18 |
| WR | - | SBSODS8 | 79 | -0.13 |  | 0.26 | 81 | 0.2 |  | 0.07 |
| WR | - | SBSODS9 | 79 | 0.36 | ** | 1.13×10^-3^ | 81 | -0.13 |  | 0.25 |
| WR | - | SBSODS10 | 79 | 0.09 |  | 0.42 | 81 | 0.03 |  | 0.8 |
| WR | - | SBSODS11 | 79 | -0.06 |  | 0.61 | 81 | 0.06 |  | 0.62 |
| WR | - | SBSODS12 | 79 | 0.01 |  | 0.9 | 81 | 0.22 |  | 0.05 |
| WR | - | SBSODS13 | 79 | -0.12 |  | 0.29 | 81 | 0.21 |  | 0.05 |
| WR | - | SBSODS14 | 79 | 0.1 |  | 0.38 | 81 | -0.1 |  | 0.39 |
| WR | - | SBSODS15 | 79 | -0.06 |  | 0.6 | 81 | 0.03 |  | 0.78 |
| WL | - | SBSODS | 79 | 0.04 |  | 0.69 | 80 | -0.1 |  | 0.39 |
| WL | - | SBSODS_emot | 79 | -0.03 |  | 0.77 | 80 | -0.21 |  | 0.07 |
| WL | - | SBSODS_funct | 79 | 0.04 |  | 0.74 | 80 | -66.2 ×10^-3^ |  | 0.96 |
| WL | - | SBSODS_neutr | 79 | 3.04×10^-3^ |  | 0.98 | 80 | -0.04 |  | 0.69 |
| WL | - | SBSODS1 | 79 | 0.03 |  | 0.77 | 80 | -43.6 ×10^-3^ |  | 0.97 |
| WL | - | SBSODS2 | 79 | 0.19 |  | 0.09 | 80 | -0.05 |  | 0.67 |
| WL | - | SBSODS3 | 79 | 0.08 |  | 0.51 | 80 | -0.14 |  | 0.22 |
| WL | - | SBSODS4 | 79 | -0.2 |  | 0.07 | 80 | -0.15 |  | 0.18 |
| WL | - | SBSODS5 | 79 | 0.02 |  | 0.87 | 80 | -0.02 |  | 0.86 |
| WL | - | SBSODS6 | 79 | -0.03 |  | 0.82 | 80 | -0.09 |  | 0.45 |
| WL | - | SBSODS7 | 79 | -0.07 |  | 0.51 | 80 | 0.08 |  | 0.5 |
| WL | - | SBSODS8 | 79 | 0.04 |  | 0.71 | 80 | -0.18 |  | 0.11 |
| WL | - | SBSODS9 | 79 | -0.02 |  | 0.83 | 80 | 0.04 |  | 0.7 |
| WL | - | SBSODS10 | 79 | -0.09 |  | 0.42 | 80 | -0.05 |  | 0.66 |
| WL | - | SBSODS11 | 79 | 0.09 |  | 0.42 | 80 | 0.02 |  | 0.83 |
| WL | - | SBSODS12 | 79 | 0.06 |  | 0.61 | 80 | -0.11 |  | 0.33 |
| WL | - | SBSODS13 | 79 | -0.02 |  | 0.86 | 80 | -0.17 |  | 0.13 |
| WL | - | SBSODS14 | 79 | 0.08 |  | 0.5 | 80 | 0.02 |  | 0.85 |
| WL | - | SBSODS15 | 79 | 0.03 |  | 0.8 | 80 | -87.7 ×10^-3^ |  | 0.94 |
| CR | - | SBSODS | 77 | 0.07 |  | 0.55 | 75 | -0.06 |  | 0.62 |
| CR | - | SBSODS_emot | 77 | 0.15 |  | 0.2 | 75 | -0.11 |  | 0.37 |
| CR | - | SBSODS_funct | 77 | 0.09 |  | 0.44 | 75 | -50.2 ×10^-3^ |  | 0.97 |
| CR | - | SBSODS_neutr | 77 | -0.05 |  | 0.66 | 75 | -0.09 |  | 0.46 |
| CR | - | SBSODS1 | 77 | 0.02 |  | 0.84 | 75 | -0.03 |  | 0.77 |
| CR | - | SBSODS2 | 77 | 0.07 |  | 0.52 | 75 | 0.04 |  | 0.73 |
| CR | - | SBSODS3 | 77 | 0.22 |  | 0.06 | 75 | -0.07 |  | 0.55 |
| CR | - | SBSODS4 | 77 | -0.02 |  | 0.86 | 75 | -0.13 |  | 0.28 |
| CR | - | SBSODS5 | 77 | 0.16 |  | 0.16 | 75 | 0.02 |  | 0.87 |
| CR | - | SBSODS6 | 77 | 0.06 |  | 0.6 | 75 | 0.02 |  | 0.89 |
| CR | - | SBSODS7 | 77 | -0.14 |  | 0.23 | 75 | 0.13 |  | 0.26 |
| CR | - | SBSODS8 | 77 | 0.26 | * | 0.02 | 75 | -0.01 |  | 0.92 |
| CR | - | SBSODS9 | 77 | -0.19 |  | 0.1 | 75 | 0.04 |  | 0.71 |
| CR | - | SBSODS10 | 77 | 0.04 |  | 0.75 | 75 | 1.01×10^-3^ |  | 0.99 |
| CR | - | SBSODS11 | 77 | 0.02 |  | 0.89 | 75 | -17.4 ×10^-3^ |  | 0.99 |
| CR | - | SBSODS12 | 77 | 0.06 |  | 0.62 | 75 | -0.13 |  | 0.28 |
| CR | - | SBSODS13 | 77 | 0.12 |  | 0.29 | 75 | -0.2 |  | 0.08 |
| CR | - | SBSODS14 | 77 | -0.09 |  | 0.43 | 75 | 0.13 |  | 0.28 |
| CR | - | SBSODS15 | 77 | 0.05 |  | 0.64 | 75 | -0.1 |  | 0.39 |
| CL | - | SBSODS | 77 | 6.39×10^-3^ |  | 0.96 | 74 | 0.05 |  | 0.69 |
| CL | - | SBSODS_emot | 77 | 0.08 |  | 0.5 | 74 | 0.18 |  | 0.12 |
| CL | - | SBSODS_funct | 77 | -0.01 |  | 0.91 | 74 | -0.01 |  | 0.9 |
| CL | - | SBSODS_neutr | 77 | 0.04 |  | 0.72 | 74 | 0.04 |  | 0.72 |
| CL | - | SBSODS1 | 77 | -0.08 |  | 0.48 | 74 | 0.12 |  | 0.3 |
| CL | - | SBSODS2 | 77 | -0.09 |  | 0.45 | 74 | 0.04 |  | 0.76 |
| CL | - | SBSODS3 | 77 | -0.2 |  | 0.09 | 74 | -59.4 ×10^-3^ |  | 0.96 |
| CL | - | SBSODS4 | 77 | 0.14 |  | 0.21 | 74 | 0.23 |  | 0.05 |
| CL | - | SBSODS5 | 77 | -0.12 |  | 0.31 | 74 | -0.07 |  | 0.58 |
| CL | - | SBSODS6 | 77 | 0.03 |  | 0.78 | 74 | 0.14 |  | 0.25 |
| CL | - | SBSODS7 | 77 | 0.06 |  | 0.58 | 74 | -0.09 |  | 0.44 |
| CL | - | SBSODS8 | 77 | 0.07 |  | 0.52 | 74 | 0.03 |  | 0.81 |
| CL | - | SBSODS9 | 77 | 0.05 |  | 0.67 | 74 | 0.03 |  | 0.83 |
| CL | - | SBSODS10 | 77 | 0.05 |  | 0.69 | 74 | 0.03 |  | 0.78 |
| CL | - | SBSODS11 | 77 | -0.04 |  | 0.74 | 74 | -0.09 |  | 0.46 |
| CL | - | SBSODS12 | 77 | -0.07 |  | 0.57 | 74 | 0.08 |  | 0.52 |
| CL | - | SBSODS13 | 77 | 0.12 |  | 0.3 | 74 | 0.16 |  | 0.17 |
| CL | - | SBSODS14 | 77 | -0.1 |  | 0.37 | 74 | -0.03 |  | 0.8 |
| CL | - | SBSODS15 | 77 | 0.01 |  | 0.92 | 74 | 8.01×10^-3^ |  | 0.95 |
| 60ms L | - | SBSODS | 81 | 0.06 |  | 0.6 | 81 | -0.13 |  | 0.24 |
| 60ms L | - | SBSODS_emot | 81 | -31.2  ×10^-3^ |  | 0.98 | 81 | -0.17 |  | 0.13 |
| 60ms L | - | SBSODS_funct | 81 | 0.08 |  | 0.46 | 81 | -0.11 |  | 0.33 |
| 60ms L | - | SBSODS_neutr | 81 | -0.03 |  | 0.8 | 81 | -0.11 |  | 0.31 |
| 60ms L | - | SBSODS1 | 81 | -0.1 |  | 0.36 | 81 | 0.16 |  | 0.14 |
| 60ms L | - | SBSODS2 | 81 | 0.21 |  | 0.06 | 81 | -0.21 |  | 0.06 |
| 60ms L | - | SBSODS3 | 81 | 0.11 |  | 0.35 | 81 | -0.08 |  | 0.45 |
| 60ms L | - | SBSODS4 | 81 | -0.14 |  | 0.23 | 81 | 0.03 |  | 0.77 |
| 60ms L | - | SBSODS5 | 81 | 0.13 |  | 0.26 | 81 | -0.03 |  | 0.76 |
| 60ms L | - | SBSODS6 | 81 | -0.09 |  | 0.45 | 81 | -0.06 |  | 0.58 |
| 60ms L | - | SBSODS7 | 81 | -0.09 |  | 0.42 | 81 | 0.1 |  | 0.38 |
| 60ms L | - | SBSODS8 | 81 | 0.12 |  | 0.3 | 81 | -0.02 |  | 0.84 |
| 60ms L | - | SBSODS9 | 81 | -0.05 |  | 0.65 | 81 | -0.01 |  | 0.9 |
| 60ms L | - | SBSODS10 | 81 | -0.06 |  | 0.57 | 81 | -0.09 |  | 0.42 |
| 60ms L | - | SBSODS11 | 81 | 0.13 |  | 0.24 | 81 | -0.06 |  | 0.59 |
| 60ms L | - | SBSODS12 | 81 | 0.04 |  | 0.75 | 81 | -0.16 |  | 0.16 |
| 60ms L | - | SBSODS13 | 81 | 0.05 |  | 0.63 | 81 | -0.25 | * | 0.02 |
| 60ms L | - | SBSODS14 | 81 | -0.03 |  | 0.77 | 81 | 0.03 |  | 0.81 |
| 60ms L | - | SBSODS15 | 81 | 0.05 |  | 0.66 | 81 | -0.11 |  | 0.31 |
| 60ms R | - | SBSODS | 81 | -41.8  ×10^-3^ |  | 0.97 | 81 | -0.11 |  | 0.35 |
| 60ms R | - | SBSODS_emot | 81 | 0.06 |  | 0.57 | 81 | -0.06 |  | 0.57 |
| 60ms R | - | SBSODS_funct | 81 | 0.06 |  | 0.6 | 81 | -0.13 |  | 0.24 |
| 60ms R | - | SBSODS_neutr | 81 | -0.07 |  | 0.51 | 81 | -0.13 |  | 0.26 |
| 60ms R | - | SBSODS1 | 81 | -0.16 |  | 0.16 | 81 | 0.18 |  | 0.1 |
| 60ms R | - | SBSODS2 | 81 | 0.09 |  | 0.4 | 81 | -0.23 | * | 0.04 |
| 60ms R | - | SBSODS3 | 81 | 0.11 |  | 0.31 | 81 | -0.13 |  | 0.25 |
| 60ms R | - | SBSODS4 | 81 | -81.6  ×10^-3^ |  | 0.94 | 81 | 0.06 |  | 0.6 |
| 60ms R | - | SBSODS5 | 81 | 0.14 |  | 0.2 | 81 | 5.28×10^-3^ |  | 0.96 |
| 60ms R | - | SBSODS6 | 81 | 0.02 |  | 0.85 | 81 | 0.01 |  | 0.89 |
| 60ms R | - | SBSODS7 | 81 | -0.15 |  | 0.18 | 81 | 0.15 |  | 0.2 |
| 60ms R | - | SBSODS8 | 81 | 0.12 |  | 0.3 | 81 | 0.04 |  | 0.7 |
| 60ms R | - | SBSODS9 | 81 | -0.14 |  | 0.2 | 81 | 0.07 |  | 0.51 |
| 60ms R | - | SBSODS10 | 81 | -0.04 |  | 0.72 | 81 | 0.02 |  | 0.86 |
| 60ms R | - | SBSODS11 | 81 | 0.05 |  | 0.64 | 81 | -0.03 |  | 0.82 |
| 60ms R | - | SBSODS12 | 81 | -0.02 |  | 0.89 | 81 | -0.11 |  | 0.34 |
| 60ms R | - | SBSODS13 | 81 | 0.07 |  | 0.51 | 81 | -0.09 |  | 0.4 |
| 60ms R | - | SBSODS14 | 81 | -0.04 |  | 0.69 | 81 | 0.01 |  | 0.91 |
| 60ms R | - | SBSODS15 | 81 | 0.06 |  | 0.61 | 81 | -0.13 |  | 0.26 |
|  | | | | | | |  | | | |
| * p < .05, ** p < .01, *** p < .001 | | | | | | |  | | | |
